# Our Children, Our Future: The Health and Well-being of First Nations Children in Manitoba, Canada.

**DOI:** 10.23889/ijpds.v7i3.1852

**Published:** 2022-08-25

**Authors:** Mariette Chartier, Wanda Phillips-Beck, Marni Brownell, Leona Star, Nora Murdock, Wendy Au, John-Michael Bowes, Brooke Cochrane, Rhonda Campbell

**Affiliations:** 1 University of Manitoba; 2 First Nations Health and Social Secretariat of Manitoba; 3 Manitoba First Nations Education Resource Centre

## Objectives

Given the impact of colonization and responding to Canada’s Truth and Reconciliation Commission, we aimed to provide baseline measures of First Nations children’s health and social outcomes in Manitoba, Canada. We also aimed to create a research process where Indigenous and non-Indigenous researchers work collaboratively and in culturally safe ways.

## Approach

We formed a team consisting of members of First Nation organizations and academic researchers. Knowledge Keepers from Anishinaabe, Cree, Anishininew, Dakota and Dene Nations guided the study, interpreted results and ensured meaningful knowledge translation. This retrospective cohort study utilized population-based health, social services, education and justice administrative data that allowed de-identified individual-level linkages across all databases through a scrambled health number. Adjusted rates and rate ratios were calculated using a generalized liner modeling approach to compare First Nations children (n=61,726) and all other Manitoba children (n=279,087) and comparing First Nations children living on and off-reserve.

## Results

Large disparities between First Nations and other Manitoba children were found in birth outcomes, physical and mental health, health services, education, social services, justice system involvement and mortality. First Nations infants had higher rates of preterm births, large-for-gestational-age births, newborn readmissions to hospital and lower rates of breastfeeding initiation compared with other Manitoba infants. Suicide rates among First Nations adolescents were ten times higher than among other adolescents in Manitoba, yet we found few differences in diagnosis of mood and anxiety disorders between the groups. First Nations children were also seven times more likely to apprehended by child protection services and youth were ten times more likely to be criminally accused. Knowledge Keepers offered their perspectives on these findings.

## Conclusion

These findings demonstrate that an enormous amount of work is required in virtually every area – health, social, education and justice – to improve First Nations children’s lives. There is an urgent need for equitable access to services, and these services should be self-determined, planned and implemented by First Nations people.

